# Identification of biological factors predictive of response to imatinib mesylate in aggressive fibromatosis

**DOI:** 10.1038/sj.bjc.6605783

**Published:** 2010-07-27

**Authors:** A Dufresne, F Bertucci, N Penel, A Le Cesne, B Bui, M Tubiana-Hulin, I Ray-Coquard, D Cupissol, C Chevreau, D Perol, A Goncalves, M Jimenez, P P Bringuier, J Y Blay

**Affiliations:** 1 Department of Medical Oncology and Department of Pathological Anatomy, Hospital Edouard Herrriot, Lyon, France; 2 Department of Medical Oncology, Biostatistics Unit, Centre Léon Bérard, INSERM U590, Lyon, France; 3 Department of Medical Oncology, Institut Paoli Calmette, INSERM UMR891, Marseille, France; 4 Centre Oscar Lambret, Lille, France; 5 Department of Medical Oncology, Institut Gustave Roussy, Villejuif Cedex, France; 6 Institut Bergonié, Bordeaux, France; 7 Centre René Huguenin, St Cloud, France; 8 Centre Val d’Aurelle, Montpellier, France; 9 Centre Claudius Regaud, Toulouse, France; 10 FNCLCC, Paris, France

**Keywords:** aggressive fibromatosis, imatinib mesylate, predictive factors

## Abstract

**Background:**

Imatinib induces responses and disease stabilisations in non-resectable patients with aggressive fibromatosis (AF). The precise target of imatinib in AF and predictive factors for response to treatment are unknown.

**Methods:**

We investigated factors potentially predictive of response to imatinib in a series of 40 patients with progressive AF included in a phase II trial of imatinib: we tested the presence of *KIT* exon 10 variant (M541L), the expression of imatinib-sensitive kinases and cell cycle proteins by immunohistochemistry (IHC), and other clinical and biological factors.

**Results:**

Of 10 patients for whom DNA could be extracted, 3 had a *KIT* exon 10 variant (30%), with no correlation with response or progression-free survival (PFS). The expression of other imatinib targets (PDGFRA/B, macrophage colony-stimulating factor receptor (M-CSFR)) and of downstream components of the cell cycle, cell proliferation and proliferation pathway (cyclin D1, ERK, MEK 1–2) did not correlate with PFS. Pre-treatment lymphopenia (<1500/*μ*l) and tumour size >120 mm correlated with shorter PFS in univariate and multivariate analyses.

**Conclusion:**

Our findings show that a baseline biological characteristic of the patient is the major parameter influencing response to imatinib in aggressive fibromatosis. Tumour characteristics, including the presence of a KIT exon 10 M541L variant, may influence tumour control but this needs to be confirmed and better explained.

Aggressive fibromatosis (AF), also known as desmoid tumour, is a rare neoplasm arising from deep musculo-aponeurotic structures. The disease, which affects 2 to 4 per 100 000 persons annually, may occur sporadically or be associated with familial adenomatous polyposis in Gardner syndrome. This slow-growing fibrous tumour may arise at any site in the body. Desmoid tumours have been classified into three main subsets, according to their location: extra-abdominal (60% of cases), abdominal wall (25%), and intra-abdominal (15%) tumours. They are considered non-malignant, with loco regional aggressiveness and a potential for recurrence but no risk of metastatic spread (Nuyttens *et al*, 2000; Enzinger and Weiss, 2001; Janinis *et al*, 2003).

When treatment is decided upon, complete surgical removal remains the best option but may be difficult or mutilating, depending on the tumour location or disease extension. Moreover, a significant proportion of patients relapse locally and/or regionally after initial surgery; in these patients, mutilating surgery and/or radiotherapy are often used, with important functional consequences in case of musculoskeletal or organ resection. When local treatment is not feasible, systemic therapies including hormonal treatments such as with anti-estrogens, administration of non-steroidal anti-inflammatory drugsor chemotherapy may induce responses, whereas some authors propose a watch and wait policy (Fiore *et al*, 2009). Imatinib mesylate (Gleevec) is a specific tyrosine-kinase inhibitor targeting kit, bcr-abl, platelet-derived growth factor receptors and, as more recently reported, M-CSFR or CSF1R. Responses and prolonged disease stabilisations with imatinib have been reported in different series of patients with relapsing AF, with 1-year progression-free survival (PFS) rates close to 60–70% (Mace *et al*, 2002; Gonçalves *et al*, 2006; Heinrich *et al*, 2006). However, the biological mechanisms underlying the cytostatic effect of imatinib in AF remain unclear.

In this work, we analysed potential predictors of response to imatinib in AF using samples from patients included in the phase II study of the French Sarcoma Group, DESMINIB (Fayette *et al*, 2007; Dufresne *et al*, 2009). The presence of the M541L KIT variant, the expression of imatinib targets and cell cycle proteins on tissue microarrays (TMAs), as well as more classical clinical and biological factors were investigated.

## Materials and methods

Patients with progressive or recurrent AF not amenable to curative surgery or radiotherapy were included in a phase II multicenter study evaluating the efficacy and toxicity of imatinib. Patients with adequate end-organ function were treated with a dose of 400 mg imatinib, increasing to 800 mg in case of disease progression. Best clinical response to imatinib (400 or 800 mg daily) was defined according to the RECIST criteria. Evaluation was carried out every 3 months. All on-treatment tumour evaluations but two were reviewed by an independent radiological review committee. Patients whose best response was complete response (CR), partial response (PR) or stable disease (SD) lasting more than 6 months were considered responsive. Patients for whom disease stabilisation lasted less than 6 months or who had disease progression were considered non-responsive. One of the secondary end points of the study was the determination of clinical and biological criteria potentially predictive of response or prolonged disease stabilisation on treatment with imatinib. Informed consent was obtained from all patients before their enrolment in the study and collection of archival pathology specimens.

In all, 40 patients were included in this study. Two patients who participated in the initial clinical study refused consent for use of their tumour samples for biological research. Paraffin-embedded tissue samples from 34 of the 40 patients (85%) were collected from the pathology centres.

### Immunohistochemistry (IHC)

A total of 5 of the 34 tissue samples were not suitable for the construction of TMAs. In all, 29 specimens were thus used as donor blocks. Sections from each donor block were stained with haematoxylin, eosin, and saffron. Morphologically representative regions were located and circled on each slide. Finally, three 1 mm cylindrical cores were extracted from the circled areas and precisely arrayed into new recipient paraffin blocks (35 × 22 × 3 mm), with a distance of 1.5 mm between cores. TMA blocks were cut into 4 *μ*m sections with a microtome.

Table 1 presents the different antibodies tested with their dilution, the duration of antigen retrieval and the duration of incubation with primary antibody. All reactions were realised using a BenchMark XT automated stainer (Ventana, Tuscon, AZ, USA). All slides were deparaffinised by heating at 75°C for 16 min. Antigen retrieval was carried out by treatment with EDTA (pH 8.0) between 30 and 60 min at 95°C. The samples were incubated for 24 or 32 min at 37°C with the primary antibody, and then 8 min at 37°C with the secondary antibody directly combined with horseradish peroxidase. All samples were counterstained with haematoxylin.

### Mutation analysis

We analysed the previously reported KIT exon 10 mutation (Gonçalves *et al*, 2006), which corresponds to an A to C point mutation (positions 1621 on mRNA and 69304 on DNA) resulting in the substitution of methionine for leucine (position 541). Genomic DNA was extracted from slides of 34 paraffin-embedded specimens using the MasterPure complete DNA and RNA purification kit (Epicentre, Madison, WI, USA). DNA of suitable quality for mutation analysis was obtained in only 10 cases, mainly because of improper fixation of paraffin-embedded tissues. Polymerase chain reaction (PCR) was used to amplify KIT exon 10. The primers used were generated by Invitrogen (Carlsbad, CA, USA): 5′-GAG TGG CTG TGG TAG AGA TC-3′ (sense) and 3′-GAG AAA GGG AAA AAT AGA TCA-5′ (antisense). PCR was carried out using Taq DNA polymerase (Transgenomic, Omaha, NE, USA) with the following cycle conditions: denaturation at 95°C, followed by 10 cycles at decreasing temperatures between 66°C and 61°C with a decrement of 0.5°C per cycle, and additional extension at 72°C. The primers used for sequencing were the same as those used for amplification. Before sequencing, PCR products were purified using Centriprep centrifugal filter device (Millipore, Billerica, MA, USA). The purified PCR product was then subjected to automated sequencing using a DNA analyser (Applied Biosystem, Carlsbad, CA, USA).

### Statistical analysis

The duration of PFS is defined as the time from the beginning of treatment to the date of disease progression or death. PFS is a continuous variable, censured at the date of disease progression or death. PFS rates were estimated using the Kaplan–Meier method and compared using the log-rank test. The Cox proportional hazards model was used to identify the factors possibly influencing the duration of PFS. Variables found significant in univariate analysis were used for multivariate analysis.

Together with protein expression and presence of KIT mutations, we assessed the following variables: gender, age, site of primary tumour, tumour size, performance status (PS), number of previous surgeries, PNN count, haemoglobin levels and lymphopenia before imatinib.

## Results

Complete clinical results of the desminib phase II study are currently being submitted for publication and will only be briefly described here. Table 2 presents the major clinical data of the study. In all, 40 patients, 12 (30%) males and 28 (70%) females, with a median age of 41 years were included in the desminib phase II trial between August 2004 and November 2005. In six cases (15%), AF was associated with familial adenomatous polyposis. Tumour sites were the limbs (*n*=24, 60%), the abdominal wall (*n*=7, 18%), the mesentery (*n*=8, 20%) and the head and neck (*n*=4, 10%). Previous treatments included surgery (*n*=33, 80%), radiotherapy (*n*=9, 23%), hormonal treatment (*n*=18, 45%), non-steroidal anti-inflammatory drugs (*n*=12, 30%), and chemotherapy (*n*=8, 20%). All patients had recurrent or progressive disease at the time of inclusion. Imatinib was given for 1 year at a dose of 400 mg/day. With a median follow-up of 34 months, PFS at 2 years was 55% (95% CI 39-69), with 4 (10%) patients in PR and 1 (2.5%) in CR (Fayette *et al*, 2007; Dufresne *et al*, 2009). After dose-escalation, 8 out of 10 patients had a second progression after transient stabilisation of their disease for a median of 12 months (range 2–30).

Immunohistochemical analysis of the expression of KIT, PDGFRA and B, M-CSFR, p42 ERK, phospho-Ser 473-Akt, phospho MEK 1–2, and cyclin D1, *β* catenin and E-Cadherin was performed on TMA. PDGFRB and *β* catenin were found expressed in all samples, cyclin D1 in 5 samples (17%) and phospho ERK in 17 (57%) without any correlation with PFS. It may be noted that none of the patients with detectable cyclin D1 expression had progressed at 1 year. No expression of M-CSFR, PDGFRA, E-Cadherin, phospho MEK 1–2, or phospho Akt on ser 473 was observed.

Among the ten patients for whom DNA was available, we observed one CR and one PR (response rate 2 out of 10, 20%) on imatinib and 8 (80%) disease stabilisations. Three (30%) tumours were found to harbour the KIT exon 10 mutation, including the patient with CR (13 months +) and two patients with SD. One PR and six disease stabilisations were observed in the other seven patients. PFS was not significantly different in patients with and without KIT mutations, nor between patients with and without DNA available for sequencing.

**Figure 1:**
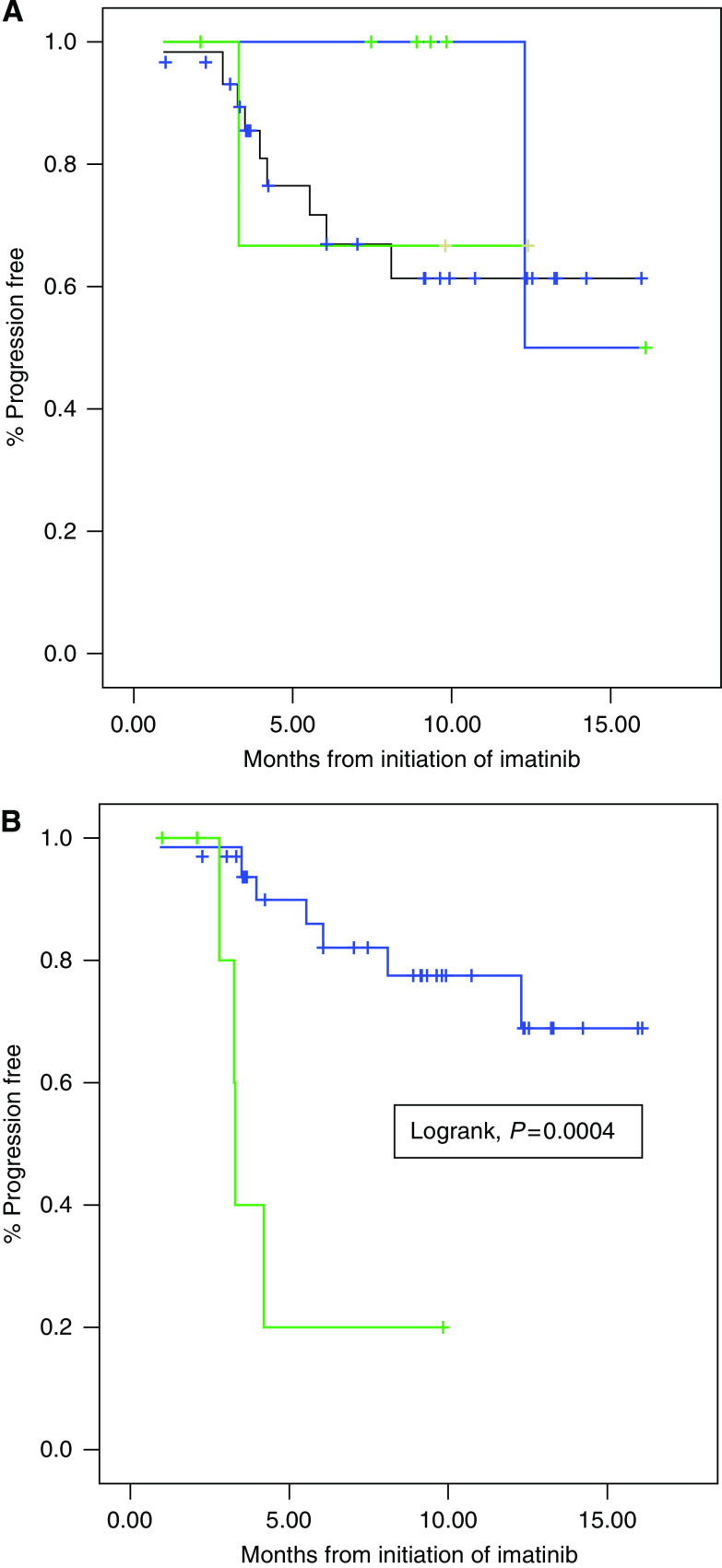
(**A**) Progression-free survival of patients with (green), without (blue) and with undetermined (black) KIT exon 10 mutation. (**B**) Progression-free survival of lymphopenic (green) and non-lymphopenic (blue) patients. (See online version for color information.)

Other clinical and biological factors were also tested for correlation with imatinib response in this series. Tumour size over 120 mm was associated with a worse PFS (median PFS, 5 months *vs* 15months, *P*=0.007). None of the other clinical characteristics tested (gender, age, site of primary tumour, PS, number of previous surgeries), nor PNN (neutrophil) count or haemoglobin levels before imatinib were found correlated with PFS. Conversely, lymphopenia (<1500/*μ*l) before initiation of imatinib was found to be highly correlated with a poorer PFS in this series (Figure 1B). Lymphopenia and tumour size above 120 mm were the only two parameters found significantly predictive of adverse outcome using the Cox model (lymphopenia HR=38, *P*=0.002; size>120 mm HR=19, *P*=0.008).

## Discussion

Several studies have failed to explain the biological mechanisms underlying the cytostatic effect of imatinib in AF. Heinrich *et al* (2006) have investigated a series of 19 patients with AF treated with imatinib and tested them for expression of several proteins (total and activated KIT, PDGFRA and B, activated PI3K Akt, MAPK and STAT3) and CTNNB1 mutations. However, they have failed to identify factors predictive of response and outcome after imatinib treatment, and no KIT expression by IHC or KIT mutation (exon 9, 11–13, 17 but not exon 10 were sequenced) has been detected. Seinfeld *et al* (2006) have reported the detection of a KIT exon 10 germline variant resulting in M541L substitution in two of four extraabdominal AF samples. Some of us have later reported on a patient with AF who responded to imatinib treatment and presented with the same KIT exon 10 variant (Gonçalves *et al*, 2006). This is the reason why we searched for M541L exon 10 variant in our series of patients treated with imatinib.

To our knowledge, this is the largest series investigating pre-imatinib tumour- and host-related molecular and clinical factors, including KIT exon 10 mutations. Even though only a limited number of cases could be investigated, response rates and PFS were not found significantly different in patients with and without the M541L KIT allele. It may be noted though, all three patients with M541L allele achieved tumour control with imatinib, including one patient with a CR. Even if not statistically confirmed, the results of the present study strongly support the clinical impression that AF patients harbouring the M541L variant are more sensitive to imatinib. The lack of statistical correlation between the presence of the M541L variant and response rate or survival is probably because of the low number of patients tested. This observation is of major interest and should be confirmed in a larger series. Moreover, the biological rationale for such a mechanism remains to be explored.

By examining cell models transfected with the exon 10 M541L variant, Tamborini *et al* (2006) and Bertucci *et al* (2007) have failed to show that the substitution could result in KIT activation or inactivation and actually corresponded to a KIT polymorphism. Both groups have concluded that the sensitivity of AF to imatinib requires an alternative explanation and possibly involves an autocrine mechanism, possibly associated with a hypersensitivity to SCF, related to the induction of a ligand-independent dimerisation induced by the exon 10 variant. The lack of correlation between expression on IHC and outcome is fully consistent with the previous report by Heinrich *et al.* Interestingly, Tabone-Eglinger *et al* (2008) have shown that GIST-type KIT mutations induce an activation-dependent alteration of normal maturation and trafficking, resulting in the intracellular retention of the activated kinase within the cell. Imatinib-induced inhibition of the phosphorylation of immature and mature mutant KIT proteins has resulted in the restoration of KIT expression at the cell surface. They conclude that these observations likely account for the absence of correlation between response to imatinib and KIT expression using IHC and may deserve to be investigated in other tyrosine kinase-activated tumours. More recently, another KIT exon 10 mutation possibly linked to imatinib response in AF has been identified, V530I (Kurtz *et al*, 2010).

The only biological parameter correlated with PFS in our patients was pre-treatment lymphopenia, whereas anaemia and PNN count had no predictive value (Figure 1A). Of note, lymphopenia was not found correlated to PS in this series. Imatinib has been previously reported to exert an anti-tumour activity in animal models through the modulation of immune response (Borg *et al*, 2004).

The present observation shows that a baseline biological characteristic of the host, not of the tumour, is the major parameter influencing response to imatinib in aggressive fibromatosis. Tumour characteristics, including the presence of the KIT exon 10 M541L variant, may have influenced tumour control in this small series but this needs to be confirmed and better explained.

## Figures and Tables

**Table 1:** Antibodies characteristics

**Antibody**	**Reference**	**Dilution**	**Ag retrieval (min)**	**Primary Ab (min)**
Anti-c-kit	A4502; Dako, Glostrup, Denmark	1 : 100	30	24
Anti-PDGFR *α*	AF 307 NA; R&D, Minneapolis, MN, USA	1 : 50	30	32
Anti-PDGFR *β*	sc-339; Santa Cruz, Santa Cruz, CA, USA	1 : 300	30	32
Anti-M-CSFR	49C10; Cell Signaling, Danvers, MA, USA	1 : 50	30	32
Anti-E cadherin	18-0223; Zymed, Carlsbad, CA, USA	1 : 70	30	24
Anti-*β* catenin	M3539; Dako	1 : 200	30	32
Anti-p42 map Kinase ERK	9108; Cell Signaling	1 : 100	30	32
Anti-phospho-AKT Ser 473	3787; Cell Signaling	1 : 50	30	32
Anti-phospho-MEK 1/2	2338; Cell Signaling	1 : 50	30	32
Anti-cyclin D1	RM-9104-R7; Neomarkers, Fremont, CA, USA	1	30	24

Abbreviations: Ab=antibody; Ag=antigen; ERK=extracellular signal regulated kinase; M-CSFR=macrophage colony-stimulating factor receptor; PDGFR=platelet derived growth factor.

**Table 2:** Patient and disease characteristics

	***N* (40)**	**%**
Age (in years) (range)	41 (20–72)	
		
*Sex*		
Female	28	70
Male	12	30
		
*PS (available for 37 patients)*		
0	27	73
1	9	24
2	1	3
		
*Tumour location (multifocal in four cases)*		
Extraabdominal	28	70
Abdominal wall	7	18
Intraabdominal	8	20
		
*Previous treatment before enrolment*		
Surgery	34	85
Radiotherapy (median dose 50 Gy)	9	23
Hormonal therapy	18	45
Treatment with NSAID	12	30
Chemotherapy	8	20

Abbreviations: NSAID=non-steroidal anti-inflammatory drug; PS=performance status.
